# Pedunculated Accessory Liver Lobe Containing the Gallbladder Presenting as Biliary Obstruction: A Case Report

**DOI:** 10.7759/cureus.88182

**Published:** 2025-07-17

**Authors:** Franco D Tarditti, Manuel B Torres, Nada A Yazigi, Carolina Rumbo, Arash R Zandieh, Gabriel E Gondolesi

**Affiliations:** 1 Medstar Georgetown Transplantation Institute, MedStar Georgetown University Hospital, Washington, D.C., USA; 2 Division of Pediatric Surgery, MedStar Georgetown University Hospital, Washington, D.C., USA; 3 MedStar Georgetown Transplantation Institute, MedStar Georgetown University Hospital, Washington, D.C., USA; 4 Department of Radiology, MedStar Georgetown University Hospital, Washington, D.C., USA

**Keywords:** accessory liver lobe, gallbladder, pediatric hepatobiliary abnormalities, pediatric hepatobiliary surgery, torsion

## Abstract

Accessory liver lobes (ALLs) are rare anatomical variations characterized by supernumerary lobes of normal hepatic parenchyma in continuity with the liver. They are typically asymptomatic and most often discovered incidentally during imaging or surgical procedures. We report a unique case of a three-month-old premature infant with a history of gastroschisis and bladder herniation, who developed clinical and biochemical signs of progressive cholestasis. Imaging studies raised suspicion for an ALL, which was confirmed intraoperatively as a pedunculated ALL in continuity with the gallbladder. The lobe was resected en bloc. ALLs are infrequently diagnosed and often present with nonspecific symptoms. While imaging may suggest the diagnosis, definitive identification is commonly made during surgery, as ALLs can mimic masses and raise concern for neoplastic disease. To our knowledge, this is the first documented case of a symptomatic ALL presenting with signs of biliary obstruction. This rare case highlights the importance of including ALL in the differential diagnosis of cholestasis, as delayed recognition may lead to secondary biliary cirrhosis, potentially necessitating liver transplantation.

## Introduction

Accessory liver lobes (ALLs) are rare anatomic variations that can arise at various sites on the liver, though they are more frequently found in the right lobe. They may present as either sessile or pedunculated, depending on their attachment to the liver [1]. ALLs consist of supernumerary lobes of normal hepatic parenchyma in continuity with the liver, distinguishing them from ectopic liver lobes, which have no anatomical connection to the main liver [1,2]. Interestingly, ALLs have been associated with ventral abdominal wall defects, raising questions about their role in abnormal liver development and hepatobiliary complications [3].

The incidence of ALLs is estimated at 0.09%, based on data from laparoscopic, open surgical, and autopsy studies conducted in Hungary and Japan [2]. However, this is likely an underestimation due to the typically asymptomatic nature of these conditions [1].

ALLs are usually discovered incidentally during imaging or surgery, often resembling liver tumors or intra-abdominal masses [1]. While most are located in the abdominal cavity, they can also occur in the thoracic cavity, complicating the differential diagnosis during initial evaluation [1,3-5].

Though frequently asymptomatic, ALLs may become clinically apparent when they cause mass effect or complications such as pedicle torsion. In such cases, they may mimic symptoms of gut ischemia, presenting with pain, vomiting, constipation, bloating, or even signs of shock. This broad range of nonspecific symptoms can make diagnosis challenging [1,3].

In this report, we present a rare case of a symptomatic ALL in a newborn with gastroschisis, emphasizing the importance of considering ALL in the differential diagnosis of cholestasis in pediatric patients. Delayed recognition may contribute to progressive cholestasis and, in severe cases, secondary biliary cirrhosis, underscoring the need for timely surgical intervention.

## Case presentation

A three-month-old premature infant, born at 33 weeks gestational age with gastroschisis and bladder herniation, underwent silo placement and sutureless closure to address the abdominal wall defect. During the postoperative course, the patient developed biochemical signs of progressive cholestasis and jaundice, prompting transfer to our hepatology and liver transplant unit.

On physical examination, a large ventral defect was noted at the umbilical site, without tenderness or rebound. The liver was palpable in the right lower abdomen. Perinatal ultrasound revealed an absent gallbladder and hepatomegaly, raising concern for a possible tumor or an ALL in the right lower quadrant, and prompting further imaging.

**Figure 1:**
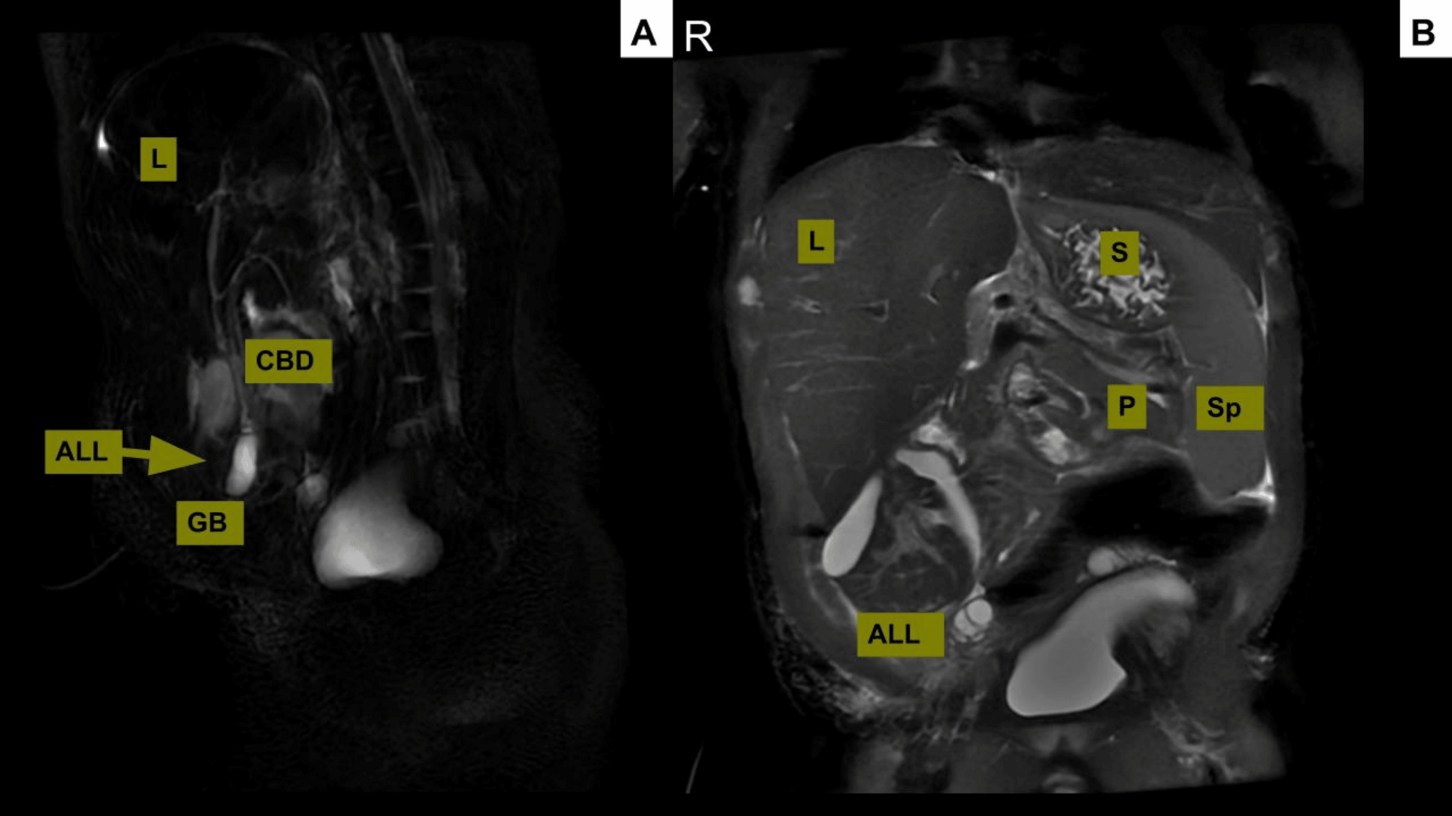
Preoperative magnetic resonance cholangiopancreatography (A) Stretching of the CBD and inferior displacement of the GB to the anterior and inferior aspect of the ALL. The GB and bile duct were transmitted through the pedunculated, pedicle-like stalk of the ALL. A second fluid-containing cystic structure resembling a gallbladder was noted at the superior aspect of the GB.  (B) Conventional T2-weighted coronal image demonstrating the ALL as a mass-like structure emanating from the right hepatic lobe. The ALL and L share isointense signal, with expected hepatic architecture including branching portal veins. The stomach, spleen, and pancreas were normal. CBD: common bile duct, GB: gallbladder, ALL: accessory liver lobe, L: liver, S: stomach, Sp: spleen, P: pancreas.

Magnetic resonance cholangiopancreatography (MRCP) was performed, confirming the presence of a ventral wall defect and identifying a perihepatic structure highly suggestive of an ALL containing a gallbladder-like structure. The MRCP also revealed marked atrophy of the right hepatic lobe, compensatory hypertrophy of the left lobe, and a cystic structure of uncertain origin. Differential considerations included a second gallbladder, a liver cyst, or an enteric duplication, though none could be definitively ruled out (Figures 1A, 1B). Table 1 summarizes the laboratory values from the day of admission to postoperative day 5.

**Table 1:** Laboratory values from admission to the day of discharge

Parameters	At admission	Preoperative	Postoperative day 2	Postoperative day 5	Reference range
Total bilirubin	10.6	7.2	3.8	2.8	0.2-1.0 mg/dL
Direct bilirubin	7.6	5.3	2.7	1.9	0-0.4 mg/dL
Aspartate transaminase	261	191	294	46	8-33 U/L
Alanine transaminase	119	90	127	40	7-55 U/L
Gamma-glutamyl transferase	674	-	-	-	5-40 U/L
Alkaline phosphatase	1026	923	531	425	30-130 U/L

Given these findings, a hepatobiliary iminodiacetic acid (HIDA) scan was performed to evaluate gallbladder function, bile flow, and overall liver function. The scan revealed hepatic dysfunction and delayed excretion of the radiotracer into the bowel. The latter was suspected to result from intermittent biliary obstruction caused by torsion of the ALL.

Based on the imaging results, the patient was referred to a specialized pediatric hepatobiliary and liver transplant unit for further evaluation and management.

After the receiving team reviewed the case, a gastrointestinal series with small bowel follow-through was requested to better understand gastrointestinal anatomy, given the known association between gastroschisis and abnormal bowel rotation. This was necessary before determining a definitive surgical approach, including the possibility of exploratory laparotomy.

The study revealed a normal duodenal course with the ligament of Treitz in an appropriate position, although an abnormal cecal location was noted. No evidence of focal stricture or mechanical obstruction was observed.

Following discussion with pediatric radiology, the exact nature of the perihepatic structure remained uncertain, although an ALL was still the leading consideration. Due to persistent clinical and biochemical signs of biliary obstruction (Table 1), the multidisciplinary team recommended exploratory laparotomy to clarify the anatomy, proceed with any necessary surgical intervention, and obtain a core liver biopsy.

**Figure 2:**
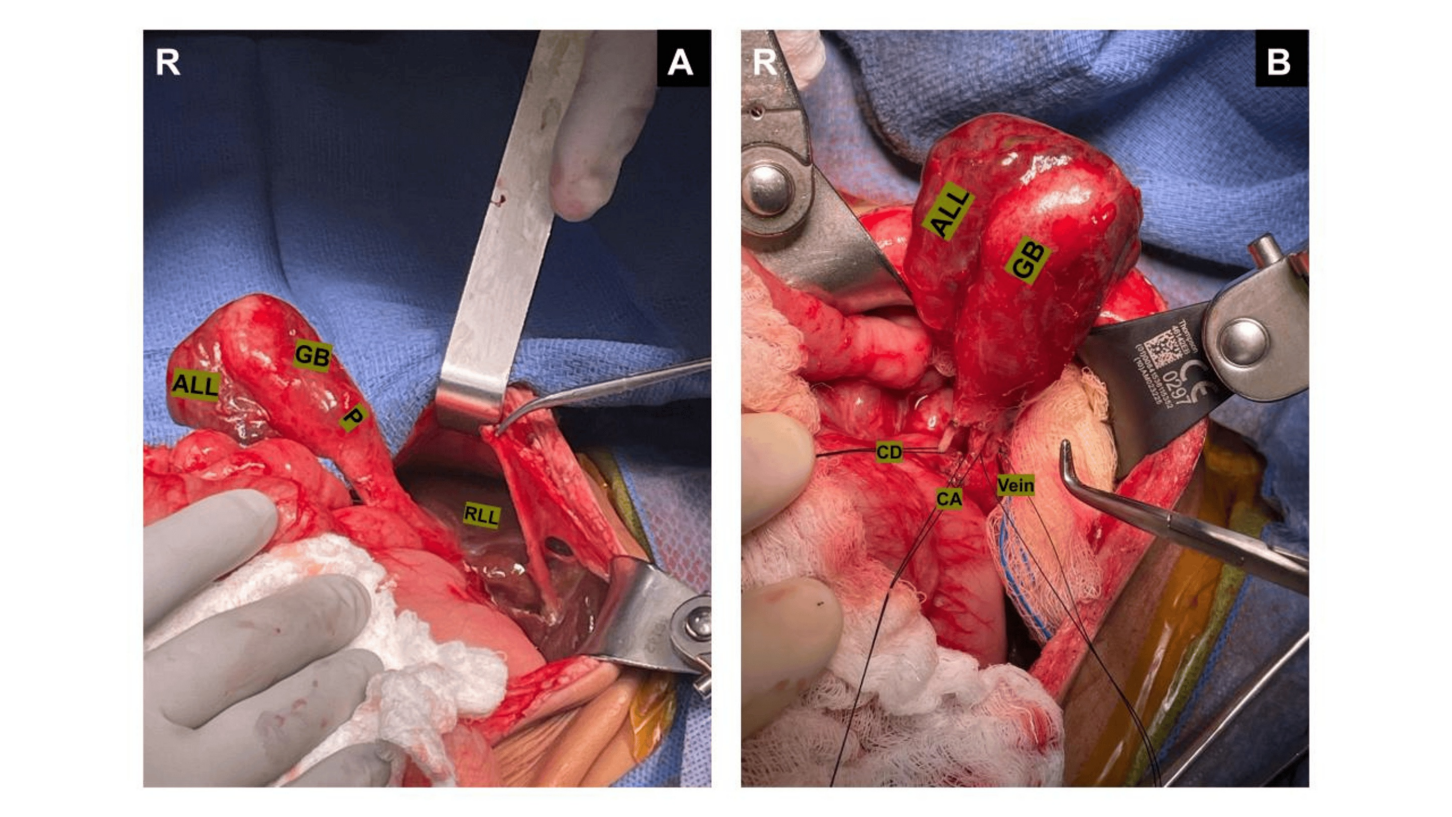
Exposure of the accessory liver lobe and the fibrous pedicle (A) Identification of the RLL and the ALL with a GB attached to the rest of the liver by a pedicle. (B) After dissection of the pedicle, three structures were exposed, identified as the vein, CA, and CD. RLL: right liver lobe, ALL: accessory liver lobe, GB: gallbladder, P: pedicle, CA: cystic artery, CD: cystic duct, R: right.

After obtaining informed consent, a midline exploratory laparotomy was performed. Intraoperatively, a pedunculated ALL was identified in the lower abdominal quadrant, extending toward the pelvis, in continuity with the common bile duct and containing the gallbladder on its anterior-inferior surface. On the posterior aspect of the lobe, an oblong subcapsular cystic lesion resembling a second gallbladder was also found (Figure 2A), accounting for the imaging findings that had suggested a duplicated gallbladder.

**Figure 3:**
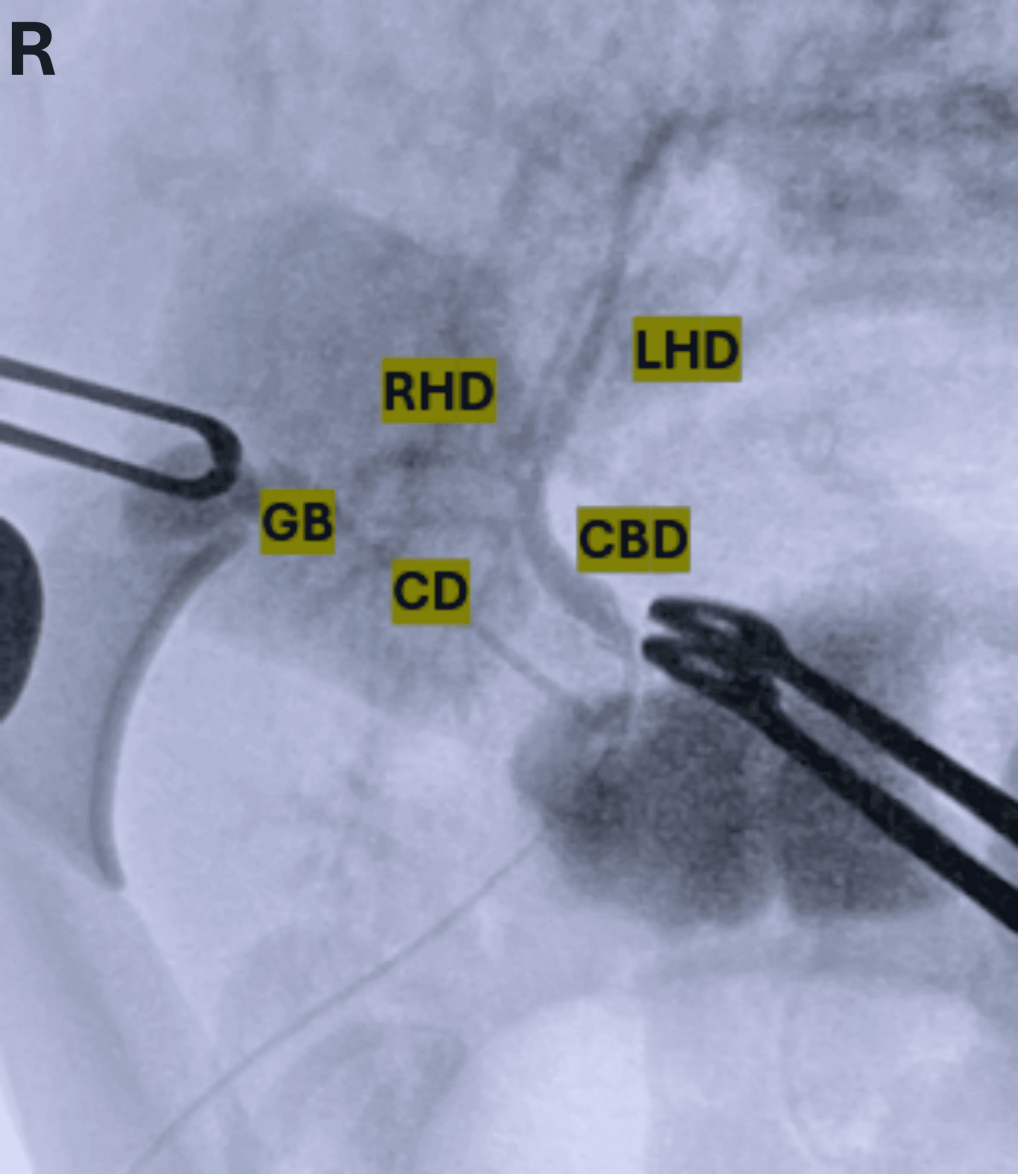
Intraoperative cholangiography showing a contrast-filled biliary tree with adequate visualization of the bile ducts The GB is draining into the CD. A small RHD and a large LHD can be seen converging into the CBD. GB: gallbladder, CD: cystic duct, RHD: right hepatic duct, LHD: left hepatic duct, CBD: common bile duct, R: right.

Attention was then turned to the remaining abdominal anatomy, with the goal of releasing intestinal adhesions and restoring normal anatomical orientation. Although the abdominal viscera were not malrotated, they were notably unattached to the retroperitoneum. After completing the lysis of adhesions, dissection of the ALL pedicle was performed. A cystic duct, cystic artery, and a significantly sized hepatic vein draining the ALL were identified (Figure 2B). Intraoperative cholangiography via the cystic duct confirmed communication with the common bile duct (Figure 3). The contrast study demonstrated a hypoplastic right hepatic duct and a prominent left hepatic duct, consistent with the segmental hypertrophy previously seen on MRCP. Contrast flowed freely into the duodenum, with no evidence of ductal dilation or filling defects.

The artery, vein, and cystic duct were individually ligated and sharply divided near the liver hilum to allow for en bloc resection of the ALL along with the gallbladder. The resected specimen measured 5.3 × 3.5 × 2.7 cm.

**Figure 4:**
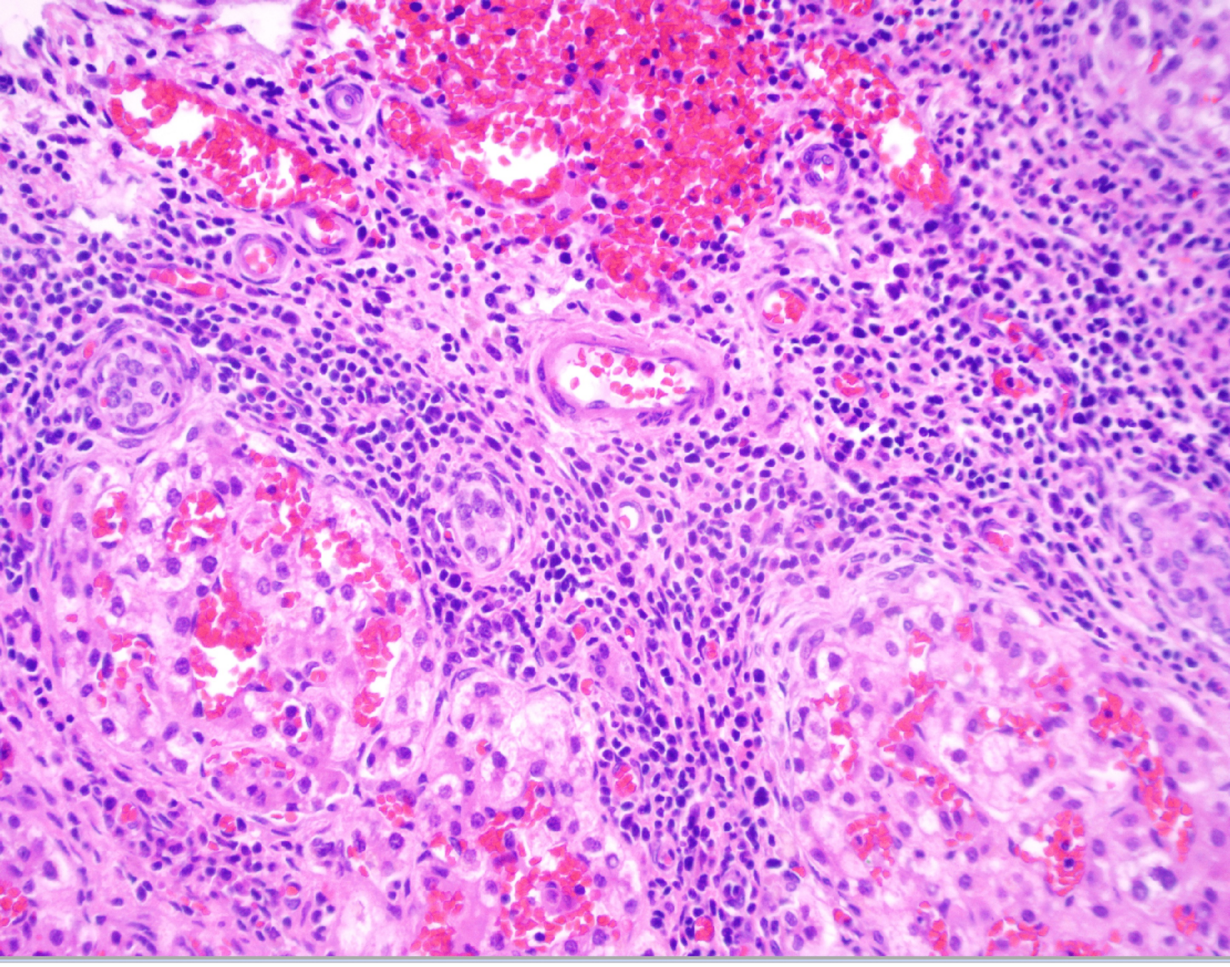
The histoarchitecture of the accessory liver lobe. Evidence of moderate-mixed portal and lobular inflammation and cholestasis admixed with extramedullary hematopoiesis.

A core needle biopsy of the right hepatic lobe was obtained using a Tru-Cut needle and submitted to pathology (Figure 4). Finally, the midline laparotomy incision was closed using interrupted 2-0 Prolene sutures, including closure of the fascial defect. A Jackson-Pratt drain was placed, and the skin was closed with 3-0 Monocryl in a transverse matrix pattern.

The postoperative course was uneventful. The patient was transferred to the PICU and remained intubated for 24 hours. On postoperative day 2, with normalization of liver function tests (Table 1) and no signs of complications, the patient was transferred to the regular pediatric unit.

By postoperative day 3, the patient had resumed bowel movements and was started on enteral feeds. The most recent liver enzyme levels showed continued improvement (Table 1). The patient was discharged on postoperative day 13.

## Discussion

ALLs are rare and usually diagnosed incidentally, as they are often asymptomatic. In some cases, patients may present with nonspecific symptoms, and there are typically no distinct physical signs. The most common presentation appears to be variable abdominal pain, often due to intermittent ischemia caused by pedicle torsion. Pérez-Martínez et al. reported a case where torsion of a pedunculated ALL led to abdominal pain, weight loss, reduced bowel movements, and projectile vomiting, making the diagnosis challenging due to a broad differential [6]. On the more severe end, Ladurner et al. described a case where torsion of a large ALL caused complete hepatic ischemia, ultimately requiring liver transplantation [7]. Pedicle torsion has also been reported during pregnancy; Fogh et al. described a case of a large pedunculated ALL that resulted in the absence of the left liver lobe and intermittent liver function abnormalities without jaundice [8]. Initially suspected to be an ovarian neoplasm, the lesion was eventually identified as a torsed ALL [8].

To the best of our knowledge, only one previous case has described a pedunculated ALL with the gallbladder in continuity in a patient with gastroschisis [9]. However, there have been no documented cases of a pedunculated ALL with a gallbladder presenting with signs of biliary obstruction, as seen in our patient.

At the time of writing, there are no reported cases of an ALL presenting with jaundice secondary to biliary obstruction. In general, the clinical presentation of ALL is unpredictable and can vary widely in both symptoms and physical findings.

Diagnosing ALL can be particularly challenging. Earlier suspicion and consideration of an ALL might have allowed the primary surgical team to remove it during the initial abdominal wall repair, potentially avoiding the need for a second surgery. However, there is limited evidence in the literature regarding the diagnostic accuracy of various imaging modalities, so most decisions are based on case reports and clinical judgment. Initial imaging workup typically includes ultrasound, CT, and MRI [1,10-14]. Ultrasound and CT can be misleading, especially if the ALL is infarcted, as the loss of internal architecture can make the mass appear hypoechoic due to congestion and reduced vascular supply [11,15]. Given these limitations, we strongly recommend confirming the diagnosis through surgical exploration, followed by biopsy to assess the histoarchitecture. While most pathology reports describe normal liver parenchyma, some have shown focal nodular hyperplasia. It's important to monitor ALLs closely due to their potential for neoplastic transformation, similar to what occurs in orthotopic liver tissue [9]. In particular, one study reported hepatocellular carcinoma arising in a normal liver alongside a noncancerous but relatively large ALL [2].

The management of ALLs remains a topic of debate, largely due to their rarity, typically asymptomatic presentation, and incidental discovery during imaging or surgery [2,5]. These congenital ectopic hepatic tissues are most often caused by embryonic heteroplasia, though they may also be acquired following trauma or surgical procedures [4,7,13]. In most cases, the volume of the ALL is small compared to the remaining liver and does not significantly affect hepatic function if removed, as shown in many published reports [14]. For instance, Wang et al. reported three cases of thoracic ALLs, one asymptomatic and two presenting as pulmonary infections, all of which were surgically resected [4].

In another case by Brandtner et al., an ALL was discovered during surgery for a small abdominal wall defect, and both were addressed in the same operation [9]. This highlights the importance of considering the removal of ALLs discovered in association with abdominal wall defects, as doing so may reduce the need for future surgical intervention and improve patient outcomes.

Currently, there is no consensus on how to manage asymptomatic ALLs. However, when symptomatic, surgical removal is strongly recommended.

## Conclusions

ALLs are rare anatomical variations that often go undetected due to their asymptomatic nature and nonspecific clinical presentation. Early recognition of ALL, especially when associated with a ventral wall defect, is crucial to prevent symptom progression, reduce unnecessary diagnostic testing, and avoid multiple surgical interventions for definitive management. While imaging protocols are largely standardized, exploratory laparotomy often remains necessary for diagnosis and treatment.

Histopathological findings in ALLs have occasionally shown features consistent with focal nodular hyperplasia, raising concerns about their long-term behavior and the need for follow-up. This case describes a pedunculated ALL containing the gallbladder, presenting with progressive intermittent cholestasis, biliary obstruction, and abdominal pain in a pediatric patient. Surgical resection should be considered the definitive treatment in similar presentations, to prevent complications such as secondary biliary cirrhosis and potential progression to liver transplant.
